# A Case Report of Biventricular Arrhythmogenic Cardiomyopathy in a Middle-Aged Female

**DOI:** 10.7759/cureus.20885

**Published:** 2022-01-03

**Authors:** Sarra Mohamed, Stephen Keane, Clare McNally, James Hayes

**Affiliations:** 1 Emergency, Fedail Hospital, Khartoum, SDN; 2 Cardiology, Cavan and Monaghan Hospital Group, Dublin, IRL; 3 Cardiology, Cavan and Monaghan Hospital Group, Cavan, IRL; 4 Respiratory and General Medicine, Cavan and Monaghan Hospital Group, Cavan, IRL

**Keywords:** cardiac arrhythmia, cardiac pacemaker, icd insertion, genetic disease, bvacm

## Abstract

Arrhythmogenic cardiomyopathy is an inherited disease in which the normal myocardium is replaced by fibroadipose infiltrates. It is increasingly being recognized as a separate entity to arrhythmogenic right ventricular cardiomyopathy though is rarely diagnosed. We report a 47-year-old female who presented to her local emergency department with a history of presyncope while driving. Electrocardiograph revealed inferolateral ST changes and right bundle branch block. A high burden of premature ventricular contractions and non-sustained ventricular tachycardia was seen on telemetry. Echocardiography showed reduced left ventricular systolic function and cardiac magnetic resonance imaging demonstrated extensive fibrosis involving the left ventricle and the septum of the right ventricle. An inherited cardiac disease genetic panel, including desmosomal gene mutations, was non-contributary. Extensive workup for other potential causes of cardiac fibrosis and reduced left ventricle function including cardiac positron emission tomography (PET) was negative. Based on the presentation and these findings, a diagnosis of biventricular arrhythmogenic cardiomyopathy was made. The patient’s condition was complicated by third-degree heart block two weeks after initiation of pharmacological treatment that included amiodarone. An implantable cardiac defibrillator was implanted. She was referred to a tertiary centre specializing in inherited cardiac conditions for familial screening.

## Introduction

Arrhythmogenic cardiomyopathy (AC) is a rare inherited disease that affects either right, left or both ventricles [1-4]. It is characterized by non-hypertrophied fibrofatty replacement of the normal myocardium [1-3]. The diagnosis of arrhythmogenic cardiomyopathy may be challenging as there are no formal criteria for diagnosis and it can be mimicked by other diseases such as cardiac sarcoidosis, dilated cardiomyopathy or amyloidosis. Unfortunately, sudden cardiac death can be the first presentation of this disease, thus, where possible patients' families should be screened for the condition [1-4].

## Case presentation

A 47-year-old female presented to the emergency department with multiple episodes of sudden onset dizziness and presyncope. The first episode occurred one week prior to her presentation while she was driving. She was able to stop her car safely and her dizziness resolved after one minute. She had further episodes of presyncope with worsening intensity and increasing duration leading to her presentation to our emergency department. She denied palpitations, shortness of breath, chest pain, or syncope. Physical examination was unremarkable. Her medical history was unremarkable and she was not on any other medications. She was an active smoker with a 10-pack-year history with minimal alcohol intake. A thorough family history did not reveal any episodes of sudden cardiac death or heart disease.

**Figure 1:**
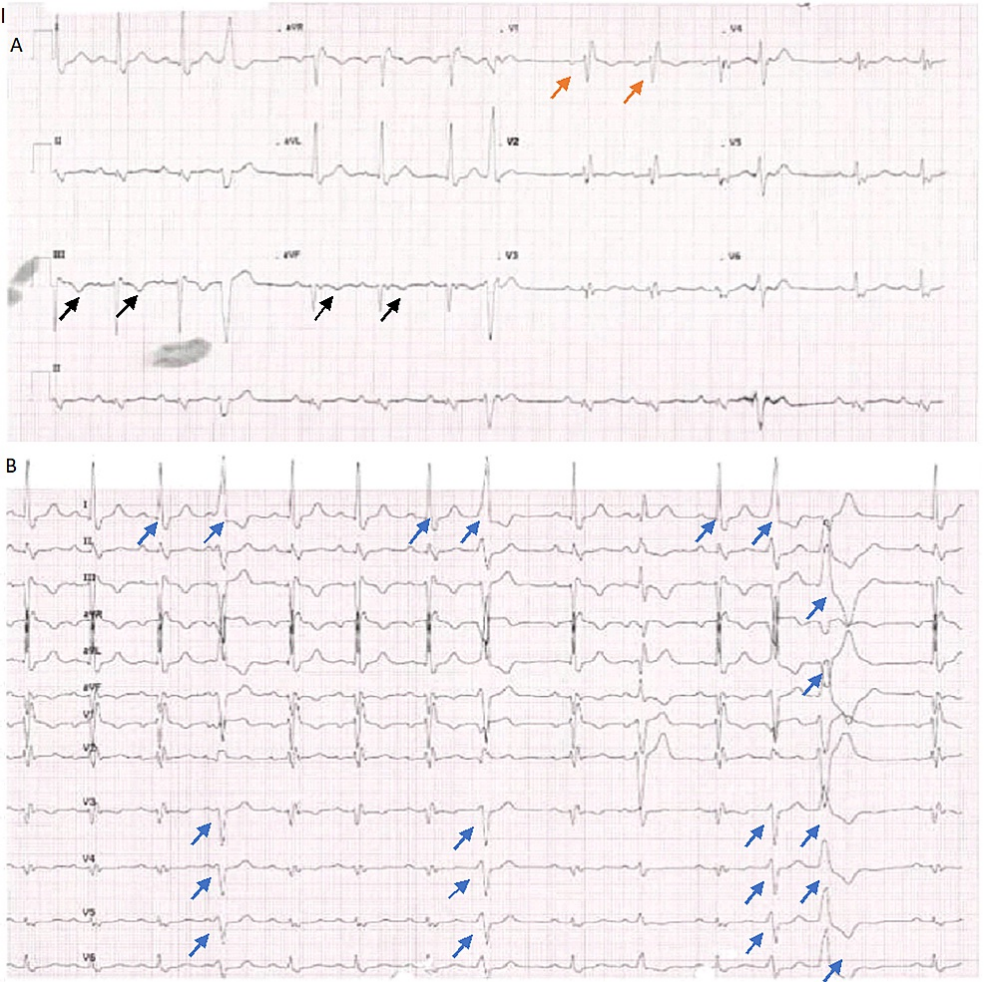
(A) ECG revealing RBBB (orange arrow) and inferior T-wave change (black arrow). (B) Frequent ectopies, bigeminy (blue arrow) ECG: Electrocardiograph; RBBB: Right bundle branch block.

**Figure 2:**
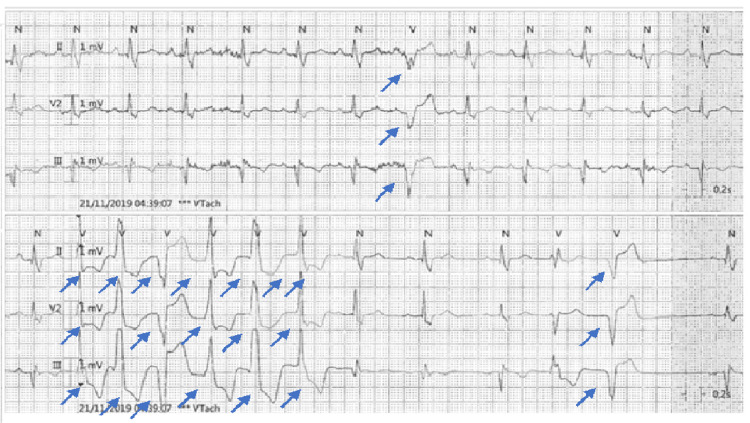
Cardiac telemetry revealing short run of non-sustained ventricular tachycardia and some ectopic beats.

**Figure 3:**
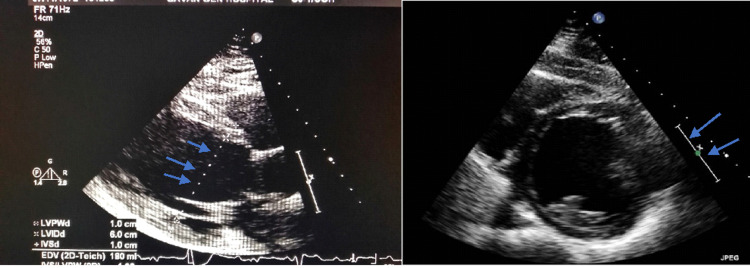
Echocardiogram showing a dilated left ventricle (arrows point to the LV dimension of 6.0 cm) with global hypokinesia. LV: Left ventricular

The initial electrocardiograph (ECG) revealed multiple ventricular ectopics, right bundle branch block (RBBB), and ST changes inferolaterally with T-wave inversion inferiorly (Figure 1). Complete blood count, urea and electrolytes, and troponin were all within normal limits. Telemetry showed a high burden of premature ventricular contractions (25%), with frequent runs of non-sustained ventricular tachycardia (Figure 2). Two-dimensional echocardiography revealed a severely reduced left ventricular (LV) systolic function (20-25%) with severe global hypokinesia and mild to moderate LV dilatation (60 mm) (Figure 3), LV wall thickness being normal.

**Figure 4:**
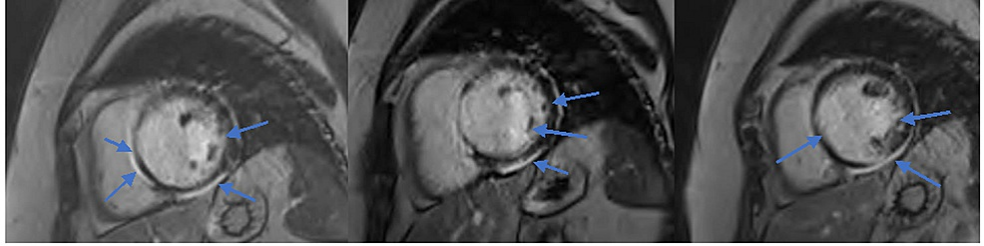
Post-contrast cardiac MRI revealing fibrosis from the base of the left ventricle to the right ventricular septum.

Cardiac magnetic radiography (MRI) cine imaging revealed global LV hypokinesia along with moderately reduced right ventricular function. Disproportionate hypokinesis in the entire septum and LV inferior wall was noted. Post-contrast cardiac MRI demonstrated extensive fibrosis from the base of the left ventricle to the right ventricular septum. It also noted prominent epicardial fibrosis throughout the inferior wall of the LV (Figure 4). These findings were consistent with arrhythmogenic cardiomyopathy with a left-sided predominance, but exclusion of sarcoidosis was recommended. She was screened for sarcoidosis through cardiac positron emission tomography (PET) scan, and sarcoidosis was ruled out. Genetic screening for desmoplakin gene mutation and other genes of inherited cardiac disease was negative. Serum angiotensin-converting enzyme and calcium level were normal. Investigations to rule out other potential causes for cardiac fibrosis - Lyme and Fabry’s disease - were unremarkable. A coronary angiogram was performed which demonstrated minimal non-obstructive coronary artery disease. The patient was diagnosed with biventricular arrhythmogenic cardiomyopathy (BVACM).

The patient was commenced on ramipril, dapagliflozin, eplerenone and amiodarone. After a discussion of the risks and benefits, an implanted cardioverter defibrillator (ICD) was arranged as an outpatient. Two weeks post-discharge, the patient represented with further dizziness and nausea. ECG and telemetry revealed intermittent complete heart block with a QTc of 525 ms. Although she was hemodynamically stable, a decision to insert a dual-chamber intracardiac defibrillator as inpatient was done successfully. She was discharged one-day post-procedure. She is being closely followed in the cardiology clinic and is awaiting an appointment in the national center for inherited cardiac conditions for familial screening.

## Discussion

The 2019 Heart Rhythm Society expert consensus statement on arrhythmogenic cardiomyopathy defines ACM as an arrhythmogenic disorder of the myocardium not secondary to ischemic, hypertensive or valvular heart disease [3]. This includes a spectrum of genetic, inflammatory, systemic and infectious disease processes [3]. The genetic form of ACM is a rare inherited disease that affects either right, left, or both ventricles [1-4]. It is characterized by non-hypertrophied fibrofatty replacement of the normal myocardium. Genetic ACM may be misdiagnosed as dilated cardiomyopathy, myocarditis, or cardiac sarcoid [1-4].

In our patient’s case, the majority of her disease was in her LV, so arrhythmogenic left ventricular cardiomyopathy (ALVC) was initially thought to be the diagnosis. ALVC is increasingly being recognized as a primary disease distinct from arrhythmogenic right ventricular cardiomyopathy (ARVC) [1,3,4]. An analysis by Miles et al. examined patients who suffered sudden cardiac death diagnosed on post-mortem as ACM, and found that 17% of cases had isolated left ventricular disease, 13% had isolated right ventricular disease and 70% had biventricular involvement [2].

While endomyocardial biopsy remains the gold standard for the definitive diagnosis it has a high false-negative rate and advances in cardiac imaging have allowed for non-invasive diagnosis [1,5].

While ALVC and ARVC share a similar disease pathology, mode of inheritance and treatment options, ALVC has some unique characteristic features [4]. These features include ECG with RBBB morphology and inferolateral ST changes, with imaging showing septal involvement and reduced LV systolic function which is generally not seen in ARVC [1,4]. Our patient demonstrated extensive septal fibrosis and marked reduction in LV systolic function along with a RBBB characteristic of ALVC. However, the imaging also revealed mildly reduced RV function, not in keeping with ALVC.

Although arrhythmogenic cardiomyopathy is considered an inherited condition, pathogenic mutations are not found in around 50% of patients [1]. Furthermore, the disease has a low penetrance rate, but certain mutations may have a high penetrance and lead to an aggressive disease course at an early age [1,2,6]. Given the negative result of the genetic panel and the unremarkable family history in our patient’s case, we consulted numerous diagnostic criteria to determine the diagnosis.

Sen-Chowdhry et al. clinical diagnostic criteria encompass the following: inferolateral T-wave changes in ECG, arrhythmia sustained or non-sustained VT, imaging findings of LV systolic dysfunction or aneurysm, and late gadolinium enhancement of the LV on cardiac MRI or fibro-adipose infiltrates on endomyocardial biopsy [4]. While our patient met these criteria for a diagnosis of ALVC, she did not meet the recent Padua criteria [3]. Published in June 2020, Corrado et al. recommended that an ACM gene mutation be identified for the diagnosis of ALVC to be established [3]. For the diagnosis of biventricular ACM, they recommend a “definite” or “borderline” diagnosis of ARVC using the 2010 revised International Task Force diagnostic criteria for ARVC with morpho-functional and/or structural LV criteria (Table 1 in the appendices) [3]. Thus, after consulting these various diagnostic criteria, we diagnosed our patient with biventricular ACM.

The management of arrhythmogenic cardiomyopathy involves managing patient's potential heart failure and life-threatening arrhythmia. Therapeutic strategies include restriction from high endurance activity, ß-blockers, antiarrhythmic and heart failure management, ICD, catheter ablation and, rarely, heart transplant [1]. In our patient's case, given the presyncope due to ventricular arrhythmia and reduced LV ejection fraction, the 2019 Heart Rhythm Society expert consensus statement on arrhythmogenic cardiomyopathy gives a class 1 indication for ICD implantation.

Though reported, the development of bradyarrhythmia is rare in the context of ACM [7]. In this case, our patient possibly developed a bradyarrhythmia with a prolonged QTc as a side effect of amiodarone. She may have been disposed to side effects of amiodarone due to fibro-adipose infiltration of the conductive system. Patients, as in our case, should be referred to a tertiary referral center that specialized in inherited cardiac conditions.

## Conclusions

We report a case of a 47-year-old lady who presented with multiple episodes of presyncope. Between the ECG, telemetry and imaging findings along with consultation with various diagnostic guidelines, she was diagnosed with biventricular arrhythmogenic cardiomyopathy. Genetic ACM remains a challenging diagnosis as it lacks standardized diagnostic criteria and in the absence of a pathogenic gene requires extensive investigation into other possible causes of ACM. This differentiation between the various forms of ACM is clinically relevant for risk stratification and familial evaluation.

## Appendices

**Table 1:** Description of clinical features and proposed diagnostic criteria of ALVC. Of note, the Padua criteria require a pathogenic gene mutation for diagnosis of ALVC. ECG: Electrocardiograph, RBBB: Right bundle branch block, Echo: Echocardiography, LV: Left ventricle, LGE: Late gadolinium enhancement, ACM: Arrhythmogenic cardiomyopathy.

	Clinical criteria proposed by Sen-Chowdhry et al.	Padua criteria (2020) proposed by Corrado et al.
ECG	Unexplained T-wave inversion in V5, V6 ± V4, I and aVL	Minor inverted T-wave in the left pericardial leads (V4-V6) Low QRS voltage
Arrhythmia	Sustained and non-sustained ventricular tachycardia of RBBB configuration document	Minor frequent ventricular extrasystoles (>500 per 24 h), non-sustained or sustained ventricular tachycardia with a RBBB morphology.
Echo	Mild LV dilatation and/or systolic impairment	Minor global LV systolic dysfunction with or without LV dilatation. Regional LV hypokinesia/akinesia of the LV free wall, septum or both
CMR	Extensive LGE of the LV myocardium (with subepicardial/mid-myocardial distribution)	Major LV LGE (stria pattern) of ≥1 Bulls’ eye segment(s) of the free wall (subepicardial/mid-myocardial), septum, or both
Family History/ Genetics		Major ACM confirmed in a first-degree relative who meets the diagnostic criteria or confirmed at autopsy or surgery. Identification of a pathogenic or likely pathogenetic ACM in the patient under evaluation. Minor history of ACM in a first-degree relative in whom it is not possible or practical to determine whether the family member meets the diagnostic criteria. Premature sudden death (<35 years) due to suspected ACM in a first-degree relative. ACM confirmed pathologically or by diagnostic criteria in a second-degree relative.
